# Effect of exenatide after short-time intensive insulin therapy on glycaemic remission maintenance in type 2 diabetes patients: a randomized controlled trial

**DOI:** 10.1038/s41598-017-02631-1

**Published:** 2017-05-24

**Authors:** Xiulin Shi, Yalin Shi, Ning Chen, Mingzhu Lin, Weijuan Su, Huijie Zhang, Changqin Liu, Haiqu Song, Fangsen Xiao, Peiying Huang, Liying Wang, Wei Liu, Jinyang Zeng, Bing Yan, Qi Liu, Suhuan Liu, Shuyu Yang, Xiaoying Li, Zhibin Li, Xuejun Li

**Affiliations:** 1 Department of Endocrinology and Diabetes, The First Affiliated Hospital, Xiamen University, Xiamen, China; 2 School of Medicine, Xiamen University, Xiamen, China; 3 Xiamen Diabetes Institute, Xiamen, China; 4 Department of Endocrinology, Zhongshan Hospital, Fudan University, Shanghai, China; 5 Epidemiology Research Unit, The First Affiliated Hospital, Xiamen University, Xiamen, China

## Abstract

Early short-term intensive insulin (STII) therapy can induce drug-free glycemic remission for up to 1 year in half of newly diagnosed type 2 diabetic mellitus (T2DM) patients. Whether exenatide following STII therapy will induce higher long-term glycaemic remission is currently unknown. To assess the effect of STII+ exenatide therapy, compared with STII only, on maintenance of glycaemic remission in newly diagnosed T2DM patients. In this randomized, parallel-group, open-label, controlled trial, 129 patients (66 in STII+ exenatide group and 63 in STII only group) firstly completed 3-week STII therapy, then STII+ exenatide group was treated with exenatide for 12 weeks further. The cumulative probabilities of 1-year and 2-year glycaemic remission in STII+ exenatide group were 68.2 ± 5.7% and 53.0 ± 6.1%, which were significantly higher than STII only group (36.5 ± 6.1% and 31.8 ± 5.9%) (p-values < 0.001). Patients in STII+ exenatide group, compared with STII only group, showed significantly decreased levels of waist (82.2 (81.0, 83.5) cm v.s. 84.2 (82.7, 85.7) cm, p = 0.048) and HbA1c (5.83 (5.60, 6.06)% v.s. 6.49 (6.20, 6.77)%, p < 0.001) after 12-week exenatide treatment, but these differences disappeared after 1-year and 2-year follow-up. As conclusions, Improved effect of sequential exenatide after STII therapy on maintenance of glycaemic remission only occurred during exenatide treatment and lost upon treatment cessation.

## Introduction

Pancreatic β-cell dysfunction is the critical pathologic process resulting in the progressive deterioration of glucose control and the complications in the natural history of type 2 diabetes mellitus (T2DM)^1^. Increasing data have shown that the early use of short-term intensive insulin (STII) therapy can improve β-cell function and induce long-term glycaemic remission in patients with newly diagnosed T2DM without further antidiabetic medication, and thus STII therapy has become a strategy of interest^2–5^. In a meta-analysis, pooling data from four studies showed that early STII therapy in the newly diagnosed T2DM patients resulted in 46% of patients remaining in 1-year drug-free remission^6^. Despite these promising results, more than half of patients receiving STII therapy will suffer hyperglycaemic relapse within 1 year.

The ideas of early STII therapy inducing long-term drug free remission in the newly diagnosed T2DM patients are to reverse β-cell deficiency and insulin resistance by rapid correction of glucotoxicity and lipotoxicity^7^. However, the generally applied STII therapy lasting 2–3 weeks is limited to correct these pathophysiological deficiencies for a long term. Therefore, new therapeutic approaches after STII therapy in the newly diagnosed T2DM patients which may improve longer term glycaemic remission are eagerly awaited.

Exenatide is the first-in-class glucagon-like peptide-1 receptor agonist (GLP-1RA) and has been proved to lower blood glucose by enhancing glucose-dependent insulin secretion of β-cells in T2DM patients^8–11^. Whether exenatide following STII therapy, compared with STII only therapy, will induce higher long-term glycaemic remission rate in newly diagnosed T2DM patients is currently unknown. Available evidence also shows that after stopping GLP-1RA treatment, their enhancement effects on β-cell function will disappear^12–14^; however, there is no evidence available on whether their effect on glycaemic remission will lose upon cessation of GLP-1RA therapy. We therefore conducted a randomized parallel-group trial with the primary aim to assess the effect of STII+ exenatide therapy, compared with STII only therapy, on maintenance of 1-year and 2-year glycaemic remission rates in newly diagnosed and drug-naive T2DM patients in China. We also aimed to test if the effect of exenatide after STII therapy on maintenance of glycaemic remission will lose upon cessation of exenatide therapy.

## Results

### Patients’ baseline characteristics

**Figure 1:**
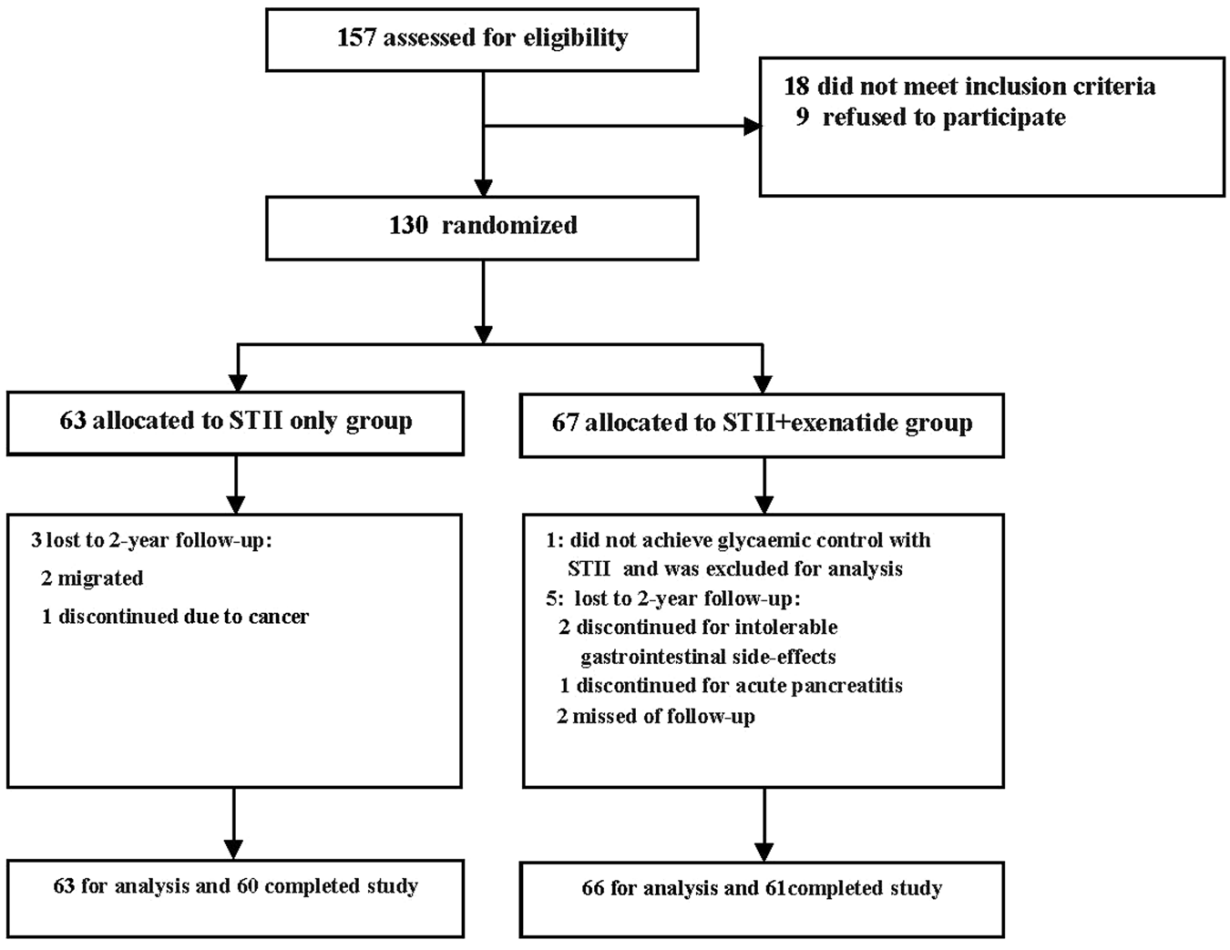
Trial profile.

Of the remaining129 subjects with the mean age of 45 ± 8 years, body mass index of 25.0 ± 3.2 kg/m^2^, FPG of 11.9 ± 3.6 mmol/L and HbA1c of 10.3 ± 2.8%, all patients in STII+ exenatide group (n = 66) and STII only group (n = 63) reached the glycaemic remission goal during STII therapy and were included for intention-to-treat analyses (Fig. 1).

Results by comparing the differences of baseline (prior to initiation of STII therapy) clinical characteristics between STII+ exenatide group and STII only group shows that glucose levels, lipid profiles, and indicates of β-cell function (HOMA-B) and insulin resistance (HOMA-IR) were comparable between the two groups (p-values < 0.05) (Table 1), but more male patients were assigned to STII+ exenatide group (87.9%) than STII only group (65.1%, p = 0.002). During STII therapy, all the 129 patients reached glycaemic remission goal with the mean days of achieving euglycaemia of 6.5 ± 2.1 days in STII only group and 6.1 ± 2.2 days in STII+ exenatide group (p = 0.444).

**Table 1:** Analysis of covariance (ANCOVA) and adjusted means (95% confidence interval (95% CI)) for laboratory and clinical parameters after the 12 weeks (after Exenatide therapy), 1 year and 2 years of STII therapy.

	STII only group		STII+ Exenatide group		P value*
	Mean/Adjusted mean	95% CI	Mean/Adjusted mean	95% CI	
Body mass index (kg/m²)					
Baseline	24.7	(23.9, 25.5)	25.2	(24.4, 25.9)	0.421
After 12 weeks	24.8	(23.7, 25.8)	23.6	(22.7, 24.4)	0.081
After 1 year	24.7	(24.1, 25.4)	24.1	(23.7, 24.5)	0.103
After 2 years	24.5	(23.7, 25.2)	23.8	(23.3, 24.3)	0.170
Waist (cm)					
Baseline	85.8	(83.6, 88.0)	87.7	(85.6, 89.8)	0.222
After 12 weeks	84.2	(82.7, 85.7)	82.2	(81.0, 83.5)	0.048*
After 1 year	84.8	(82.4, 87.2)	82.7	(81.1, 84.3)	0.151
After 2 years	82.7	(80.3, 85.1)	81.0	(79.3, 82.6)	0.247
Systolic blood pressure (mm Hg)					
Baseline	122.9	(120.3, 125.6)	124.4	(122.0, 126.9)	0.083
After 12 weeks	125.7	(122.4, 129.0)	121.9	(119.3, 124.6)	0.082
After 1 year	130.2	(124.4, 136.0)	124.7	(120.8, 128.6)	0.127
After 2 years	128.4	(121.9, 135.0)	125.0	(120.5, 129.5)	0.404
Diastolic blood pressure (mm Hg)					
Baseline	77.8	(75.6, 78.0)	79.2	(77.5, 81.0)	0.312
After 12 weeks	79.4	(77.0, 81.8)	78.0	(76.0, 80.0)	0.374
After 1 year	78.4	(74.0, 82.9)	77.2	(74.2, 80.2)	0.654
After 2 years	73.7	(69.5, 77.9)	74.4	(71.5, 77.4)	0.784
Triglyceride (mmol/L)					
Baseline	2.38	(1.57, 3.21)	2.09	(1.65, 2.53)	0.516
After 12 weeks	1.17	(0.93, 1.42)	1.11	(0.91, 1.31)	0.707
After 1 year	1.38	(0.89, 1.87)	1.55	(1.16, 1.93)	0.602
After 2 years	1.30	(0.88, 1.71)	1.32	(1.07, 1.58)	0.923
Total cholesterol (mmol/L)					
Baseline	5.12	(4.84, 5.40)	5.15	(4.86, 5.48)	0.326
After 12 weeks	4.70	(4.23, 5.17)	4.21	(3.81, 4.61)	0.119
After 1 year	4.45	(4.05, 4.86)	4.72	(4.39, 5.03)	0.316
After 2 years	4.83	(4.37, 5.29)	4.75	(4.46, 5.03)	0.768
HDL-cholesterol (mmol/L)					
Baseline	1.03	(0.92, 1.37)	1.26	(1.06, 1.47)	0.416
After 12 weeks	1.29	(1.03, 1.55)	1.36	(1.14, 1.58)	0.670
After 1 year	1.12	(0.91, 1.33)	1.37	(1.20, 1.54)	0.072
After 2 years	1.11	(0.59, 1.63)	1.67	(1.36, 1.99)	0.073
LDL-cholesterol (mmol/L)					
Baseline	3.17	(2.88, 3.46)	3.30	(3.10, 3.55)	0.158
After 12 weeks	2.87	(2.53, 3.21)	2.28	(1.98, 2.56)	0.009*
After 1 year	2.70	(2.34, 3.07)	2.65	(2.36, 2.94)	0.817
After 2 years	3.11	(2.56, 3.65)	2.50	(2.15, 2.85)	0.066
Fasting plasma glucose (mmol/L)					
Baseline	12.1	(11.3, 13.1)	11.2	(10.5, 12.4)	0.226
After 12 weeks	6.32	(5.85, 6.78)	6.31	(5.93, 6.69)	0.984
After 1 year	5.97	(5.54, 6.41)	6.08	(5.80, 6.37)	0.678
After 2 years	6.02	(5.45, 6.67)	6.26	(5.85, 6.68)	0.604
HbA1c (%)					
Baseline	10.2	(9.59,10.84)	9.9	(9.27,10.49)	0.601
After 12 weeks	6.49	(6.20, 6.77)	5.83	(5.60, 6.06)	< 0.001*
After 1 year	6.25	(5.90, 6.59)	6.17	(5.92, 6.41)	0.701
After 2 years	6.16	(5.50, 6.82)	6.11	(5.66, 6.57)	0.912
HOMA-B					
Baseline	45.3	(35.2, 55.4)	44.0	(35.6, 52.3)	0.842
After 12 weeks	89.4	(57.1, 121.8)	77.3	(51.9, 102.7)	0.564
After 1 year	123.9	(87.5, 160.4)	84.6	(59.5, 109.6)	0.083
After 2 years	95.9	(71.6, 120.2)	85.0	(70.9, 99.0)	0.446
HOMA-IR					
Baseline	2.93	(2.51, 3.34)	2.83	(2.39, 3.27)	0.746
After 12 weeks	2.82	(2.25, 3.39)	2.87	(2.43, 3.32)	0.872
After 1 year	3.42	(2.64, 4.19)	3.04	(2.50, 3.58)	0.435
After 2 years	3.55	(2.79, 4.30)	2.68	(2.25, 3.11)	0.051

*p < 0.05, p value for analysis of covariance (ANCOVA) between two groups with adjustment for the corresponding variables at baseline when necessary.

HDL, high-density lipoprotein; HOMA, homeostasis model assessment; IR, insulin resistance index; LDL, low-density lipoprotein cholesterol.

### Maintenance of 1-year and 2-year glycaemic remission

Within 12-month follow-up period after STII therapy, 47 patients in STII+ exenatide group and 25 patients in STII only group sustained in glycaemic remission. The cumulative probability of maintenance of 1-year glycaemic remission in the STII+ exenatide group was 68.2 ± 5.7%, which was significantly higher than that of STII only group (36.5 ± 6.1%) (log-rank test: p < 0.001). During 24-month follow-up, 37 patients in STII+ exenatide group and 22 patients in STII only group maintained glycaemic remission, and the cumulative probability of maintenance of 2-year glycaemic remission in the STII+ exenatide group was 53.0 ± 6.1%, which was also significantly higher than that of STII only group (31.8 ± 5.9%) (log-rank test: p < 0.001).

**Figure 2:**
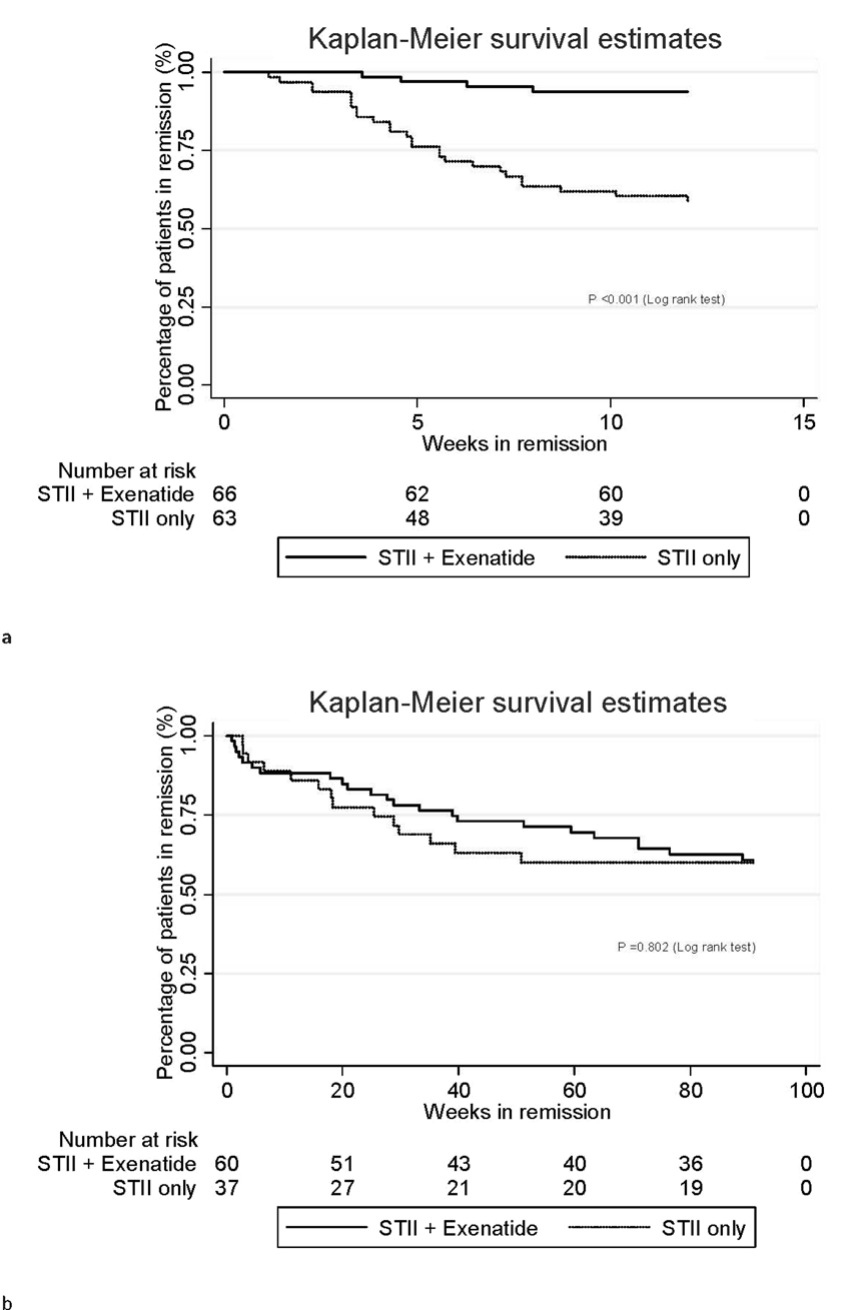
Cumulative probability of remission (**a**). During the 12-week exenatide treatment after STII therapy; (**b**). From the 13th week to 2 years of follow-up after STII therapy.

The duration of the whole 24-month follow-up after STII therapy was further divided into two separate parts: Part I: the first 12 weeks of follow-up after STII therapy, which was the duration of exenatide treatment for STII+ exenatide group; and Part II: from the beginning of the 13^th^ week (after cessation of exenatide treatment for STII+ exenatide group) to the end of 24 months of follow-up. During Part I follow-up, the cumulative probabilities of glycaemic remission maintenances were 90.9 ± 3.5% for the STII+ exenatide group and 60.3 ± 6.2% for the STII only group (log-rank test: p < 0.001) (Fig. 2a). And Fig. 2b showed that the cumulative probabilities of glycaemic remission between the two groups during Part II follow-up were not statistically significant (log-rank test: p = 0.802).

### Differences of repeated measurements

Repeated measurements on clinical parameters, blood pressure, lipid profiles, FPG, HbA1c, insulin resistance (HOMA-IR) and basal β-cell function (HOMA-B) were conducted for patients sustained in glycaemic remission only. After 2 days of exenatide cessation in STII+ exenatide group and 12 weeks after insulin cessation in STII only group, patients in both groups, compared with their baseline levels, had significantly decreased levels of body mass index, waist, FPG, HbA1c, triglycerides, LDL-C and significantly increased levels of HOMA-B (data not shown). As for differences between the two groups, analyses of covariance with adjustment for corresponding values at baseline showed that patients in STII+ exenatide group (N = 60), compared with those in STII only group (N = 37), had significantly decreased levels of waist (marginal adjusted means [95%CI]: 82.2 [81.0, 83.5] v.s. 84.2 [82.7, 85.7], p = 0.048), LDL-c (2.28 [1.98, 2.56] v.s. 2.87 [2.53, 3.21], p = 0.009) and HbA1c (5.83 [5.60, 6.06] v.s. 6.49 [6.20, 6.77], p < 0.001) (Table 1). But differences of FPG, HOMA-IR and HOMA-B were not statistically significant between the two groups. As for the repeated measurements after the 12th month and 24th month of follow-up, analyses of covariance with adjustment for corresponding values at baseline showed all the differences between STII+ exenatide group and STII only group were not statistically significant (Table 1).

### Adverse effects and tolerability

During STII therapy, there were no severe hypoglycaemic episodes defined as an event requiring the assistance of another person to actively administer carbohydrate, glucagon, or other resuscitative treatments, and minor hypoglycaemic episodes defined as having classical symptoms of hypoglycaemia or blood glucose level below 3.1 mmol/L and prompt recovery after the patient self-administered carbohydrate was similar between the two groups (14/66 in STII+ exenatide group v.s. 12/63 in STII only group, p > 0.05). For exenatide treatment, the most frequently observed adverse event was mild-to-moderate nausea (48.4%); and the other gastrointestinal adverse events included vomiting and fatigue. Two patients (3.0%) withdrew as a result of nausea or vomiting. One patient in STII+ exenatide group developed pancreatitis, which was resolved after withdrawal of the exenatide medication. One patient in STII only group developed esophagus cancer and was given surgical operation after withdrawal.

## Discussion

The present study found that, for newly diagnosed and drug-naive T2DM patients who reached glycaemic remission by STII therapy, sequential exenatide therapy for 12 weeks induced significantly higher 1-year and 2-year maintenance of glycaemic remission rates compared with STII only therapy. However, this improvement on maintenance of glycaemic remission only occurred during the 12-week exenatide treatment and was completely lost after stopping exenatide treatment. Furthermore, the effects of exenatide on lowering waist and HbA1c during exenatide therapy totally disappeared upon exenatide cessation. Thus, the beneficial effects of sequential exenatide therapy for 12 weeks after STII on maintenance of glycaemic remission and lowering waist and HbA1c did not sustain upon cessation of exenatide treatment.

T2DM is characterised by β-cell dysfunction and insulin resistance, and glucotoxicity and lipotoxicity, which are effects of elevated glucose and free fatty acids on β-cell mass and function, have been proved to have deleterious effect on both β-cell function and insulin action^15^. Insulin has been shown to protect the β-cell by inducing rapid reversal of glucotoxicity and lipotoxicity^16^, and short-term treatment with insulin therapy has been proposed for eliminating glucolipotoxicity and thus has been become a strategy of interest. Chinese patients with T2DM generally have severe β-cell dysfunction at diagnosis, and preservation of β-cell function is an more important treatment goal in newly diagnosed Chinese T2DM patients^17^. Although STII therapy has been shown to improve β-cell function and induce long-term glycaemic remission^6^, there were still more than half of patients who suffered hyperglycemic relapse within 1 year, and new strategies aimed to improve longer term maintenance of drug-free glycaemic remission after STII are therefore warranted.

Studies in cultured β-cells and rodent models have consistently shown that GLP-1RA treatment may help preserve beta-cells function. However, evidence about the effect of GLP-1RA, e.g. exenatide, following STII therapy on long term glycaemic remission rate is not available. Our results demonstrate that sequential exenatide treatment for 12 weeks after STII therapy can induce significantly higher maintenance of 1-year and 2-year glycaemic remission rates as compared to STII only therapy. The present study also found that, during exenatide treatment, patients in STII+ exenatide group had significantly decreased levels of HbA1c, LDL-c and waist as compared with those in STII only group. Therefore, one possible reason for the higher maintenance of 1-year and 2-year glycaemic remission rates in the STII+ exenatide group compared with STII only group is that STII can eliminate glucolipotoxicity, improve β-cell function and thus achieve optimum glycaemic control, and sequential exenatide treatment following STII may maintain or further enhance β-cell function and then improve the maintenance of glycaemic remission. Reduction of waist during exenatide therapy may contribute to another possible reason for the improved maintenance of glycaemic remission in STII+ exenatide group, because body fat reduction per se has been shown to improve β-cell function in subjects with and without type 2 diabetes^18, 19^.

Retnakaran and colleagues reported that after the initial improvement in β-cell function achieved with pre-randomisation intensive insulin therapy, GLP-1RA liraglutide induced a robust further enhancement of β-cell function that was sustained for 48 weeks in early T2DM but lost upon cessation of therapy^13^. Bunck MC and co-workers also found that exenatide significantly improved β-cell function during 1-year of treatment in T2DM patients, but β-cell function and glycemic control returned to pretreatment values after cessation of therapy^20^. However, after following a longer period (3-year) treatment with exenatide, they further reported that the disposition index, another β-cell function index, was sustained after a 4-week off-drug period^21^, which may imply that a sustained beneficial effect on β-cell preservation may be induced with longer treatment with exenatide. However, evidence on whether the effect of GLP-1RA on glycaemic remission will disappear upon treatment cessation is not available now. The present study found that the improvement of 1-year and 2-year maintenance of glycaemic remission rates in STII+ exenatide group mainly occurred during the 12-week exenatide treatment. After cessation of exenatide treatment, the cumulative probabilities of maintenance of glycaemic remission between the two groups were not statistically significant. Furthermore, we also found the effects of exenatide on lowering waist and HbA1c for patients in STII+ exenatide group during exenatide treatment disappeared upon cessation of exenatide therapy. Therefore, our data, together with others, suggests that sequential exenatide for 12 weeks after STII therapy may not be enough to maintain beneficial effect on glycaemic remission and that ongoing treatment of GLP-1RA, e.g. exenatide, or further treatment with other methods should be explored and emphasized.

Several limitations of the present study need to be recognized. First, our sample size was not enough (130 eligible patients were randomly assigned into two groups while a total of 144 patients were needed based on power calculation) and all subjects were recruited from one hospital only, which may hampered the generalization of our findings. Second, repeat measurement on clinical and laboratory tests were available for patients sustained in glycaemic remission only but not for those with hyperglycemic relapse, which resulted in uncompleted data, and therefore we might underestimate differences of repeat measurements on clinical parameters during follow-up between these two groups. Third, these patients had relatively higher baseline HbA1c levels (around 10%), representing a group with poor glycaemia control. So our results may not necessarily be translated to the common patients whose HbA1c levels are lower than 10%. Last but not least, our results may be biased due to the following limitations: (a) more males patients were allocated into STII+ exenatide group, which may imply our randomization procedure might not be conducted adequately; (b) our study was not blinded to patients, although the nurses and medical students who collected data were not aware of patients’ allocation; (c) patients may change their life style habits during the 2-year follow-up period, but this may not confound our results much. These limitations, however, do not diminish the value of this study. For example, our study is, to the best of our knowledge, the first to apply a therapeutic strategy of sequential exenatide after STII therapy in newly diagnosed and drug-naive T2DM patients. Second, the total 2 years of follow-up duration was relatively long enough.

In conclusion, the present study demonstrated that sequential exenatide therapy for 12 weeks for newly diagnosed and drug-naive T2DM patients who reached glycaemic remission goal by STII therapy can induce significantly higher maintenance of 1-year and 2-year glycaemic remission rates as compared to STII only therapy, but the improvement on maintenance of glycaemic remission mainly occurred during the 12-week exenatide treatment and was completely lost after stopping exenatide treatment. Therefore, our results suggest that effect of exenatide after STII therapy for 12 weeks on maintenance of glycaemic remission did not sustain upon cessation of exenatide treatment and that ongoing treatment of exenatide or further treatment with other methods should be emphasized. For future study that may address further the remission effect of STII therapy in newly diagnosed T2DM patients, sequential treatment with incretin-related agent (GLP-1RA and dipeptidyl peptidase- 4 (DPP- 4) inhibitors^22^ or the sodium-glucose cotransporter 2 (SGLT2) inhibitor^23^, actively compared with metformin or placebo, should be explored.

## Methods

### Design and setting

This study was designed as a randomized, parallel-group, open-label, controlled trial. One hundred and fifty-seven patients, who were newly diagnosed with T2DM according to WHO criteria (1999) and had not received any antihyperglycaemic therapy, were screened in 2013 from the First Affiliated Hospital of Xiamen University, Xiamen, China. Eligibility criteria for this trial included age 30–70 years, fasting plasma glucose (FPG) 7.0–16.7 mmol/L, and body mass index (BMI) 20.0~35.0 kg/m^2^. Patients with the following conditions were excluded: acute or severe chronic diabetic complications, maturity onset diabetes in youth, mitochondria diabetes mellitus, severe intercurrent illness, or positive for glutamic acid decarboxylase antibody.

The study aimed to explore effect of exenatide after STII therapy on maintenance of 1-year glycaemic remission was registered with Clinical Trials. gov (number NCT01776788) with 1 year of follow up after STII therapy. To explore effect of exenatide after STII therapy on longer time glycaemic remission, extension of follow-up period for another 1-year after STII therapy was registered with Chinese Clinical Trial Registry (www.chictr.org.cn, number ChiCTR-IPR-15006298). Written informed consent was obtained from all patients before undergoing randomisation. The study was approved by the human ethics committees of the First Affiliated Hospital of Xiamen University and conducted in compliance with Good Clinical Practice (GCP) guidelines and the Declaration of Helsinki.

### Subjects and treatment

Of the 157 patients screened, 18 did not meet the inclusion criteria and 9 refused to participate in the trial. Then 130 eligible patients were randomly assigned to STII+ exenatide group (n = 67) or STII only group (control group, n = 63). The randomisation was conducted independently at a central office using a computer-generated random allocation sequence table. Allocation concealment was performed by enclosing assignments in sequentially numbered, opaque, closed envelopes.

There was a 7-day run-in period of diet and physical exercise only. Then all patients received STII treatment in the hospital: insulin (Recombinant Human Insulin Lispro Injection, Lilly, US) with an insulin pump (Medtronic Minimed Paradigm Insulin Pump). Initial insulin doses were 0.4–0.5 IU/kg and total daily doses were divided into 50% of basal and 50% of bolus injection. Patients were monitored of capillary blood glucose levels at least six times a day, enabling titration of insulin doses to attain the glycaemic remission goal, which was defined as a fasting capillary blood glucose of 4.0–6.0 mmol/L and capillary blood glucose at 2-h after each of three meals of less than 8.0 mmol/L throughout the period of STII therapy. One patient who did not reach glycaemic remission goal was regarded as requiring additional or different therapy and was excluded from the efficacy analysis. All of the left 129 patients in two groups (n = 66 in STII+ exenatide group and n = 63 in STII only group) reached the glycaemic remission goal, and STII treatment was maintained for 2 weeks further. Then patients were discharged from the hospital, and patients in the STII+ exenatide group were trained and treated sequentially with exenatide (Exenatide Injection, AstraZeneca) at home. Exenatide treatment was initiated at a dose of 5 ug b.i.d. (injected 30 min before breakfast and dinner) for 4 weeks, and followed at a dose of 10 ug b.i.d. for 8 weeks. Patients in the STII only group did not receive any further treatment after STII therapy.

### Measurements and endpoints

Baseline characteristics, including socio-demographic status, present and previous health history and medications, were collected during face-to-face interview by the trained nurses and medical students who were blinded to study objectives. Anthropometric measurements were obtained using standard protocols and techniques. After removal of shoes and heavy clothing, each subject underwent weight, height and waist circumference measurements, using a calibrated scale. Arterial blood pressure was measured with a mercury sphygmomanometer after sitting for at least 15 minutes. Three readings were taken at 5-min intervals. The mean of the three measurements was recorded.

Fasting blood samples after a 10-h overnight fasting were used to measure FPG, glycosylated hemoglobin A1c (HbA1c), fasting insulin and lipid profiles. For conducting 2-h postprandial plasma glucose (2h-PPG) test, all patients were provided with the standardized breakfast which contained 439 kcal (caloric contribution: 47% carbohydrate, 31% fat, and 22% protein) after a 10-h overnight fasting. All laboratory tests were done in the central laboratory of the First Affiliated Hospital of Xiamen University. FPG and 2h-PPG were measured by using the hexokinase method, insulin by radioimmunoassay (3 V Bio-engineering group, Weifang, China), and HbA1c by the Bio-Rad Variant Hemoglobin A1c assay. Homoeostasis model assessment (HOMA) was used to estimate basal β-cell function (HOMA-B) and insulin resistance (HOMA-IR) with the following equations: HOMA-B = 20*fasting serum insulin (Flns, mU/ml)/(fasting blood glucose (FPG, mmol/L)-3.5), and HOMA-IR = fasting serum insulin (Flns, mU/ml) *fasting blood glucose (FPG, mmol/L)/22.5.

Follow-up for maintenance of glycaemic remission was initiated after STII therapy for both groups. After exenatide treatment for STII+ exenatide group and insulin treatment for STII only group, patients in both groups were instructed to continue diet and physical exercise only. Maintenance of glycaemic remission was ascertained by systematic glycaemic monitoring (FPG and 2h-PPG) monthly during the initial 3 months and at a 3-month interval thereafter. All patients were asked to conduct self-monitoring of blood glucose at home and encouraged to contact the medical staff with any intercurrent problem. Maintenance of glycaemic remission was defined as FPG < = 7.0 mmol/L or 2h-PPG < = 10.0 mmol/L. Hyperglycaemia relapse was defined as either FPG > 7.0 mmol/L or 2h-PPG > 10.0 mmol/L, and confirmation of hyperglycaemia relapse was conducted two week later. The time of hyperglycaemic relapse was recorded and patients with hyperglycaemia relapse were treated with metformin and then gliclazide-MR for the remaining study period according to the guideline of Chinese Diabetes Society.

The primary endpoints were the maintenance of 1-year and 2-year glycaemic remission rates which were calculated from the time point after STII therapy for the original study and the extension study, respectively. Secondary endpoints were repeat measurements of clinical parameters (including FPG, HbA1c, basal β-cell function (HOMA-B), insulin resistance (HOMA-IR), etc) during follow-up.

### Statistical analysis

The original study with 1-year duration of follow-up was designed as a superiority trial aimed to show STII+ exenatide group was superior to the STII only group on maintenance of 1-year glycaemic remission. Our previously pilot study in 2010 showed that around 70% (11 of 15) patients who received exenatide after STII achieved 1-year remission; and existing evidence showed that 45% patients who received STII only achieved 1-year remission^6^. The margin of an acceptable difference of 1-year drug-free remission rate between two groups was set as 25%. The criterion for establishing superiority was that the lower bound of the two side 95% confidence interval (CI) for the difference between the two groups must exceed the predefined superiority margin of 5%. Under these assumptions, a sample size of 65 patients in each of the 2 treatment groups was needed to yield 80% power to conclude that STII+ exenatide group was superior to the STII only group (control group) with α = 0.05. Further taking account of the dropout rate as around 10%, then 72 patients was needed for each group. And the extension study was further extended for another 1-year of follow-up after STII therapy.

Data were analyzed using Stata14.0 (StatCorp, College Station, TX) and performed on the intention-to-treat population. Differences of baseline characteristics between the two groups were compared using analyses of variance for continuous variables and chi-square test for categorical variables. For differences of repeat measurements between these two groups after treatment (2 days after exenatide cessation in STII+ exenatide group and 12 weeks after insulin cessation in STII only group, 1 year and 2 years after insulin cessation for both groups), analyses of covariance were performed with adjustment for corresponding values at baseline. Kaplan-Meier plots were used to analyse the time for the 1-year and 2-year maintenance of glycaemic remission from the time point after STII therapy, which were compared between two groups with the use of log-rank test. Significance levels were defined as p < 0.05.

### Clinical Trial Registration Numbers

NCT01776788, www.ClinicalTrials.gov, Registration date: January 22, 2013; ChiCTR-IPR-15006298, www.chictr.org.cn, Registration date: April 21, 2015.
